# Concept confirmation of the Treatment Administration Satisfaction Questionnaire (TASQ) in rare paroxysmal nocturnal hemoglobinuria

**DOI:** 10.1186/s41687-021-00319-9

**Published:** 2021-06-21

**Authors:** Helen Doll, Ufuk Coşkun, Chris Hartford, Ioannis Tomazos

**Affiliations:** 1 Clinical Outcomes Solutions Ltd, Basepoint, Shearway Road, Shearway Business Park, Folkestone, Kent CT19 4RH UK; 2 Clinical Outcomes Solutions Ltd, 1820 E. River Rd, Suite 220, Tucson, AZ 85718 USA; 3 Alexion Pharmaceuticals Inc., 121 Seaport Blvd, Boston, MA 02210 USA

**Keywords:** Paroxysmal nocturnal hemoglobinuria, Eculizumab, TASQ-IV, TASQ-SC, Ravulizumab

## Abstract

**Background:**

This study was conducted to evaluate content validity of the IntraVenous and SubCutaneous Treatment Administration Satisfaction Questionnaires (TASQ-IV and TASQ-SC), for use in a clinical trial population of participants with paroxysmal nocturnal hemoglobinuria (PNH) undergoing eculizumab treatment.

**Methods:**

Participants underwent semi-structured combined brief introduction to disease history and full cognitive debriefing interviews to establish symptoms and key impacts of PNH and to explore the clarity and relevance of both sets of instructions (TASQ-IV and TASQ-SC). The clarity, relevance, response options, and recall period of the TASQ-IV items were also explored.

**Results:**

Ten participants with PNH were recruited. Fatigue was the most commonly reported symptom (*n* = 7); the most commonly reported impact of PNH was on physical activity (*n* = 4). Nine participants indicated understanding and relevance of the TASQ-IV instructions; three participants suggested changes. Of the 20 TASQ-IV items, ≥ 15 were considered understandable, relevant and to have suitable response options (*n* ≥ 8). The TASQ-SC instructions were understood by all participants; seven participants indicated relevance. While a few participants suggested minor changes for the items, these reflected the one-off completion of the measure in an interview setting and were thus not considered sufficient to justify modification of the measure for clinical trial completion.

**Conclusions:**

Most participants understood the TASQ-IV and TASQ-SC instructions (*n* = 9 and 10, respectively) and the TASQ-IV items were considered clear, relevant and to have suitable response options, demonstrating face and content validity of the instruments for the clinical trial setting.

**Supplementary Information:**

The online version contains supplementary material available at 10.1186/s41687-021-00319-9.

## Introduction

### Burden of PNH

Paroxysmal nocturnal hemoglobinuria (PNH) is a rare and potentially life-threatening hematologic disorder [1–5]. PNH is characterized by intravascular hemolysis, resulting in symptoms due to uncontrolled activation of the terminal complement pathway, leading to intravascular hemolytic anemia, prothrombotic state, end organ damage, and increased risk of morbidity and mortality [1–6]. PNH is a clinically heterogeneous disease, with varying symptoms at initial presentation, which may include significant fatigue or weakness, shortness of breath, hemoglobinuria, abdominal pain, suppression of bone marrow production, thrombosis, and renal insufficiency [5, 7]. The prevalence of PNH is estimated to be 1–1.5 cases per million in the USA [8] and 15.9 cases per million in Europe [9].

### Eculizumab treatment of PNH

Eculizumab (Soliris®; Alexion Pharmaceuticals, Inc., Boston), a humanized monoclonal antibody that inhibits complement terminal C5 activation, was the first regulatory-approved treatment for PNH [10]. Eculizumab has been shown to reduce intravascular hemolysis and associated clinical complications [11] and is effective and well tolerated [12]. More recently, ravulizumab (Ultomiris®; Alexion Pharmaceuticals, Inc., Boston), a long-acting C5 inhibitor, has been approved for the treatment of PNH in the USA (December 2018) and several other countries [13, 14]. Clinical trials have demonstrated that ravulizumab is as safe and efficacious as eculizumab and is associated with reduced rates of breakthrough hemolysis compared with eculizumab [14].

### TASQ-IV and TASQ-SC measures of treatment satisfaction

The Treatment Administration Satisfaction Questionnaire (TASQ) is a patient-reported outcome measure of satisfaction with and impact of treatment, which was developed from the Rituximab Administration Satisfaction Questionnaire (RASQ) [15, 16]. The measure has been shown to have face and content validity and good psychometric properties [17]. There are two forms of the questionnaire: the TASQ – Intravenous (TASQ-IV) and the TASQ – Subcutaneous (TASQ-SC).

The TASQ consists of 19 items that are used to rate treatment satisfaction, pain, redness and swelling, side effects, anxiety, worry about condition worsening, confidence in treatment, restrictions due to IV (or SC within the TASQ-SC), administration, convenience, duration of treatment administration, impact on daily activities, time gained or lost due to treatment, time to talk to nurses or doctors about disease, preference for IV or SC, and likeliness of recommending IV (or SC within the TASQ-SC) treatment administration. All ratings are made using a 5-point Likert scale.

### Hypothesis and objectives

The study hypothesis was that the TASQ-IV and TASQ-SC tools are face and content valid for clinical trial use in a patient population with PNH undergoing eculizumab treatment. The objective of this study was to conduct cognitive debriefing interviews to evaluate the face and content validity of the TASQ-IV and TASQ-SC in patients with PNH with specific reference to its use in clinical trials.

## Methods

### Participants

Participants were recruited over a three-month period through a patient advocacy group, Aplastic Anemia and MDS International Foundation (AA-MDS). A short description of the study was posted in a closed forum with Clinical Outcomes Solutions’ (COS) contact information. Participants emailed COS to indicate interest and were screened by phone for eligibility. The first 10 participants who met the eligibility criteria (Supplementary Table 1) were included in the study; all were actively receiving eculizumab treatment. As PNH is a rare disease, there was no target recruitment quota.

All participants provided informed consent and completed demographic and case report forms.

### Semi-structured interview: introduction to disease history

This part of the interview was conducted in approximately 5–10 min, with the aims of building rapport and establishing a conversation for the cognitive debriefing.

### Semi-structured interview: cognitive debriefing

Cognitive debriefing is a structured interview technique commonly carried out in the development of patient-reported outcomes to test a questionnaire or instrument among individuals from a target population. This includes examining whether patients understand the question wording and recall period, interpret the questions correctly, and use the response scale appropriately. The participants were also provided with the opportunity to suggest changes to the questions or response options to strengthen the measure, and were asked at the end of the interview for relevant details of any relevant content related to their experience of PNH considered to be missing from the questionnaire.

Cognitive debriefing was conducted in approximately 65 min. This included debriefing of the TASQ-IV instructions and individual items and the TASQ-SC instructions (Supplementary Material).

For the TASQ-IV, participants were asked to discuss their understanding, the relevance of, and any suggested improvements for the instructions and items. In addition, they were also asked to discuss the response options and recall period for the individual items. A coding scheme was used to score statements, with participants providing a ‘yes’, ‘no’, or ‘maybe’ response.

For the TASQ-SC, participants were asked to discuss understanding and relevance of the instructions and any suggested improvements. Only the instructions were debriefed for the TASQ-SC because the items in this questionnaire are very similar to that of the TASQ-IV, namely that “intravenous” is replaced with “subcutaneous”. Additionally, as participants had not been previously treated with the SC formulation, feedback on such items (e.g. recall period) was considered not relevant.

### Analysis process

This study utilized both deductive and inductive reasoning to interpret qualitative data within a thematic analysis framework [18]. The analysis began with open-ended coding of transcribed data which allowed emerging of theme (inductive approach), where participants discussed their diagnosis, symptoms, health-related quality of life impacts, and overall impressions of the measure. Themes were then refined and updated using an iterative process. Due to time limitations, data coded for the inductive approach was limited to 5–10 min of the 75-min-long interviews. For the cognitive debriefing a deductive approach was taken as many of the codes were pre-selected (understanding/relevance of item concepts, suitability of recall period/response options, and participant suggestions).

Following the participants’ feedback, decisions on whether changes were needed to the TASQ-IV/TASQ-SC instructions and the TASQ-IV items were made based on the context in which the feedback was given, the number of participants suggesting a change, the intended use of the instrument in a clinical trial setting, and the rare disease status of PNH. In particular, any changes suggested by only one participant were considered unlikely to affect the general validity of the measure.

## Results

### Demographics

Ten participants (six women and four men) with a mean age of 51.7 ± 15.3 years were recruited and included in the study (Table 1). All participants were of white/Caucasian ethnicity. Seven participants (70%) had a college/university degree or higher indicating a population with high educational ability.

**Table 1:** Demographic characteristics of participants in the content validity study of the TASQ-IV and TASQ-SC measures

Demographic characteristic	Statistic (***N*** = 10)
**Age (years)**	
*N*	10
Mean (SD)	51.7 (15.3)
Median (IQR)	53 (39–65)
Range	28–74
**Sex**	
Female	6
Male	4
**Race**	
White/Caucasian	10
**Education**	
High school diploma	1
Some college/certification	2
College/university degree	2
Graduate degree	5
**Work status**	
Employed full-time	4
Employed part-time	1
Retired	2
Unemployed	1
Other	2

Categorical variables were described by the frequency and percentage of each response choice, with missing data being included in the calculation of percentage. Continuous variables were described by their frequency, mean, standard deviation, median, quartiles 1 and 3, extreme values (minimum and maximum values) and frequency and percentage of missing values

*IQR* interquartile range, *IV* intravenous, *PNH* paroxysmal nocturnal hemoglobinuria, *SC* subcutaneous, *SD* standard deviation, *TASQ* Treatment Administration Satisfaction Questionnaire

### Time since diagnosis, comorbidities, and misdiagnoses

**Fig. 1:**
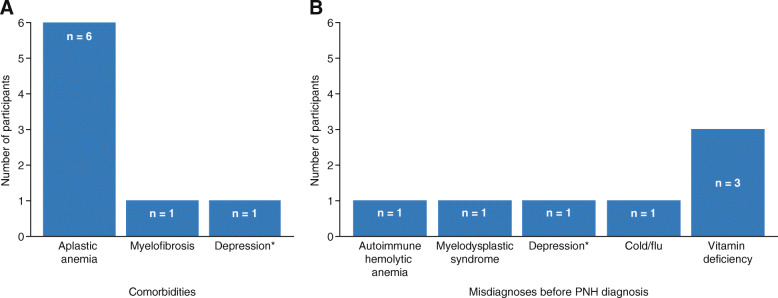
Participant-reported comorbidities (**a**) and misdiagnoses before PNH diagnosis (**b**). *PNH* paroxysmal nocturnal hemoglobinuria. *Depression was mentioned by two different participants as a comorbidity or misdiagnosis

The mean time since PNH diagnosis was 9.2 ± 6.3 years (range, 0.5–20 years). The most commonly reported comorbidity was aplastic anemia (*n* = 6), followed by myelofibrosis (*n* = 1) and depression (*n* = 1; Fig. 1a). Vitamin deficiency was the most commonly reported misdiagnosis (*n* = 3); other misdiagnoses included autoimmune hemolytic anemia (*n* = 1), myelodysplastic syndrome (*n* = 1), depression (*n* = 1), and cold or flu (*n* = 1; Fig. 1b).

### Symptoms and impacts of PNH

**Fig. 2:**
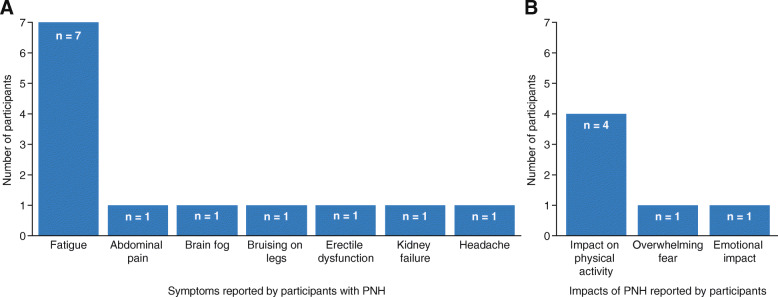
Participant-reported symptoms (**a**) and impacts of PNH (**b**) *PNH* paroxysmal nocturnal hemoglobinuria

The most commonly reported symptom of PNH was fatigue (*n* = 7; Fig. 2a). The most commonly reported impact of PNH was on physical activity (*n* = 4); PNH was also reported to result in overwhelming fear (*n* = 1) as well as having an emotional impact (*n* = 1) (Fig. 2b).

### Participant opinions and suggestions for the TASQ-IV instructions

Nine participants demonstrated understanding of the TASQ-IV instructions and thought the items were relevant, with one participant’s response unclear (counted as “maybe understood” and “maybe relevant”).

Of three participants who suggested changes to the TASQ-IV instructions, two had confirmed their understanding of the instructions (Supplementary Table 2). The suggested changes included adding a port option to the instructions, because some participants receive eculizumab treatment via a port: *“they should probably add the port option. I think people will be confused and it’s an option in one of the later questions.”* (Participant 01-007). Omitting the detailed description of IV treatment and mentioning SC treatment as an option were also suggested (Supplementary Table 3). However, changes to the measure were not recommended for the following reasons: only one participant suggested each of these changes; in a clinical trial setting the participants would receive training in how to complete the measure (e.g., for a port option); and recording detailed description of the IV treatment would be useful to those who need it.

### Participant opinions on the TASQ-IV items and the overall measure

**Fig. 3:**
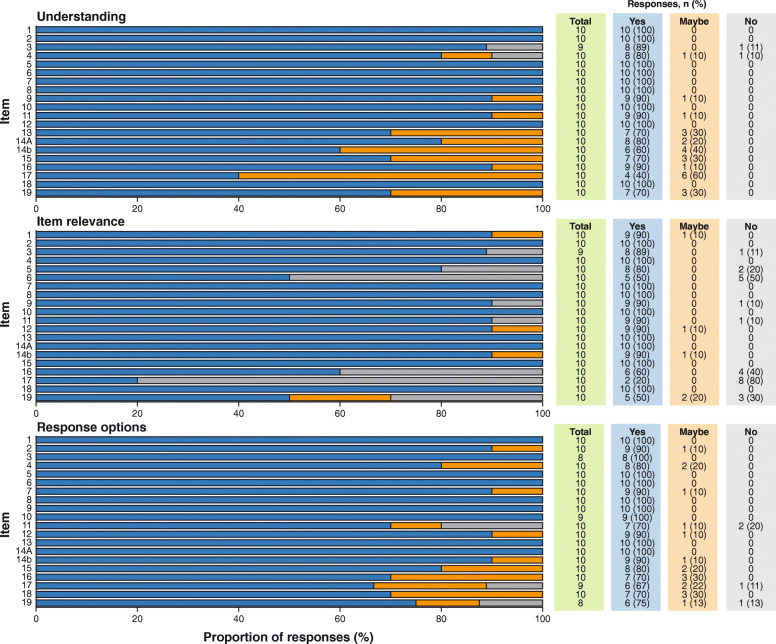
Participant responses regarding the TASQ-IV items by item debrief; understanding, item relevance and response options

**Fig. 4:**
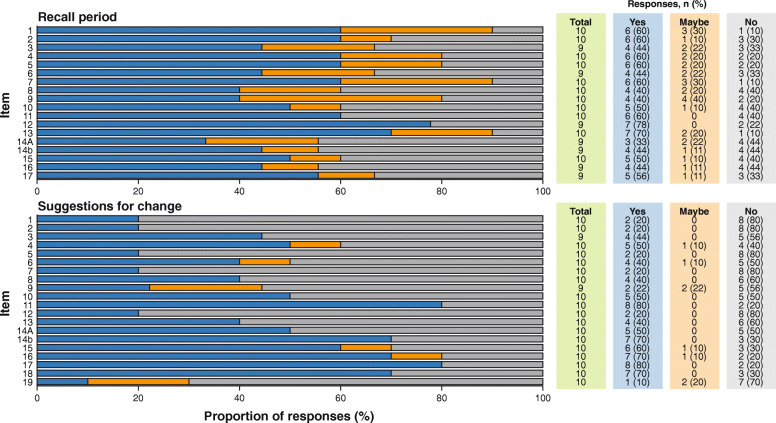
Participant responses regarding the TASQ-IV items by item debrief; recall period and suggestions for change

Eight or more participants indicated that the items were understandable (15 items) and relevant (16 items) and that the response options were suitable (15 items) (Fig. 3), while the recall period for each item was considered suitable by fewer than eight participants (Fig. 4).

Item 17 (impact of IV infusion on the amount of time to talk to nurse and/or doctor) received the lowest number of responses regarding item relevance, with only two participants indicating that this item was relevant.

### Suggested changes to the TASQ-IV items

#### Item 1 – satisfaction or dissatisfaction with the IV infusion

Two changes to item 1 were suggested: lengthening the recall period (*n* = 1) and defining satisfaction and dissatisfaction (*n* = 1). *“Well, it’s asking about the infusion, well again, someone might want to separate out, you know, in more detail. Did you feel better the same day? Did you feel better the next day? Did he feel better for the next 10 days or 14 days?...were you satisfied or dissatisfied?...”* (Participant 02-010).

#### Item 2 – pain, swelling, or redness at the injection site

Participants suggested two changes to item 2: expanding the body area from injection site to the whole arm (*n* = 1) and measuring redness, pain, and swelling separately (*n* = 1).

#### Item 3 – pain during the IV process

Four changes to item 3 were suggested: changing the item to the past tense (*n* = 1), lengthening the recall period (*n* = 1), mentioning the recall period in the item (*n* = 1), and differentiating the IV infusion process from the experience afterward (*n* = 1). *“…actually, say whether you’re talking about pain from the actual medication going into your body or are you talking about symptoms that you may experience afterwards.”* (Participant 01-008).

#### Item 4 – side effects

Six changes to item 4 were suggested: changing the item to the past tense (*n* = 1), adding the word “process” after the IV infusion (*n* = 1), differentiating the first IV experience from the last IV experience (*n* = 1), lengthening the recall period (*n* = 1), adding possible side effects (*n* = 1), and comparing the severity of side effects to any physician-described expectations (*n* = 1). *“I suppose you could say…‘…or were the side effects…better or worse than your doctor led you to believe?’...or something like that.”* (Participant 01-004).

#### Item 5 – anxiety about the IV infusion

Two changes to item 5 were suggested: changing the item to the past tense (*n* = 1) and lengthening the recall period (*n* = 1).

#### Item 6 – worrying during the IV infusion that the condition could get worse

Four changes to item 6 were suggested: changing the item to reflect that the drug itself is not making the condition worse (*n* = 2), differentiating the IV process from the drug (*n* = 1), lengthening the recall period (*n* = 1), and broadening the item question to ask about overall health instead of just PNH (“condition”; *n* = 1). *“I would definitely…broaden the question because…there’s definitely some more to PNH that you have to worry about, not just the PNH. I guess condition could be changed to overall health.”* (Participant 01-009).

#### Item 7 – feeling anxious during the IV infusion thinking about the disease

Two changes to item 7 were suggested: differentiating the IV process from the drug (*n* = 1) and changing the question into the past tense (*n* = 1).

#### Item 8 – confidence in the IV treatment

Six changes to item 8 were suggested: changing the question to the past tense (*n* = 1), lengthening the recall period (*n* = 1), clarifying whether the item is about the treatment or the drug (*n* = 1), stating the name of the drug in the item question (*n* = 1), mentioning “confident” in each response option (*n* = 1), and asking recall “before treatment” (*n* = 1). *“I would say it would be best to ask before treatment, so on the second treatment, but before…if you ask it after, you’re not gonna get a real response, because they’re gonna be getting the medicine. And I know my before and after, if you were to ask me that question of my last, last IV, would be completely different.”* (Participant 01-009).

#### Item 9 – feeling restricted by the IV infusion

Five changes to item 9 were suggested: changing the item to the past tense (*n* = 1), making the item more specific (*n* = 1), specifying the type of restriction (*n* = 1), having response options refer to the item (*n* = 1), and clarifying the exact time frame of restriction (during the infusion vs. day of infusion; *n* = 1). *“And…maybe it’s meant to be vague, you know?...‘...Do you feel restricted by the infusion,’ again I didn’t know if they meant um, physically or schedule, or is it you know, impacting your life? I wasn’t quite sure. So, I just looked at it exactly as the exact infusion appointment and being you know, tethered to the IV for a couple hours.”* (Participant 01-002).

#### Item 10 – convenience of the IV infusion

Four changes to item 10 were suggested: separating home from clinic-based infusions (*n* = 2) removing one of the response options (“inconvenient”; *n* = 1), combining items 9 and 10 (*n* = 1), and clarifying the time frame (drip time versus total visit time; *n* = 1). *“I think they should specify here whether they mean the actual amount of time it takes to infuse...or complete time. I put ‘too long,’ because…when I think about how long it takes to get Soliris, I don’t really think about infusion time. I think about…how long it takes the pharmacy to get it to the nurse so that the nurse can hook me up.”* (Participant 01-008).

#### Item 11 – amount of time to have the IV infusion

Four changes to item 11 were suggested: clarifying the time period regarding the IV infusion (during infusion compared with whole time between infusions; *n* = 4), removing a response option (too short; *n* = 3) changing the item question into past tense (*n* = 1), and eliminating the item (*n* = 1).

#### Item 12 – expected length of time to have the IV infusion

One change to item 12 was suggested by two participants, which was to clarify the time period regarding the IV infusion (during infusion compared with entire visit/travel time), for example: *“Again, I would say, distinguish between the drip time versus the entire visit, because historically, before, not including the length and time, which I’ve only had a couple of few times over the years, 35-minute infusion is not the majority of time involved in the whole process... Even at my last infusion center where I could be an hour and a half...”* (Participant 01-001).

#### Item 13 – bother by the time of the infusion

Two changes to item 13 were suggested: clarifying the time period regarding the IV infusion (during infusion compared with whole time between infusions; *n* = 3); one participant suggested lengthening the recall period.

#### Item 14A – interfering with daily activities

Five changes to item 14A were suggested, clarifying the time period regarding the IV infusion (during infusion compared with whole time between infusions; *n* = 3), changing the question to past tense (*n* = 1), differentiating item 14A from item 14B more clearly (*n* = 1), clarifying the item question (*n* = 1), and lengthening the recall period (*n* = 1). *“I think overall…is a little more relevant to say does it typically interfere or thinking of the past two or three months of infusions.”* (Participant 01-004).

#### Item 14B – limiting daily activities

Five changes to item 14B were suggested: clarifying the time period regarding IV infusion (during infusion compared with whole time between infusions; *n* = 2), eliminating the item (*n* = 2), lengthening the recall period (*n* = 2), adding the recall period to the item (*n* = 1), and clarifying the response option “Always” (*n* = 1). *“…[Response option] ‘always’ is absolute, right? Meaning…is there something I absolutely, cannot, possibly do?...”* (Participant 01-006).

#### Item 15 – losing or gaining time due to the IV infusion

Participants suggested six changes to item 15: clarifying the time period regarding IV infusion (during infusion compared with whole time between infusions; *n* = 2), asking for longer recall in addition to the last treatment (*n* = 1), removing one of the options (*n* = 1), making the item more nuanced (*n* = 1), changing the item wording from “apply the infusion” to “receive the infusion” (*n* = 1), and changing a phrase to make it easier to read (*n* = 1). “*…‘because of the length of time to do’..., I found that ‘apply the IV infusion’ to be strange language... For ‘the length of time to’... I don’t know. It’s weird. ‘Length of time required for the IV infusion.’”* (Participant 01-004).

#### Item 16 – talking to a nurse/doctor during the IV infusion

Seven changes to item 16 were suggested: lengthening the recall period (*n* = 1), shortening response option statements (*n* = 1), taking the word “nurse” out of the item question and response options (*n* = 1), separating the item question for each nurse and doctor (*n* = 1), changing the item to ask about the IV process and not illness (*n* = 1), clarifying the item (*n* = 1), and adding new response options (*n* = 2). *“The choice of ‘I had too much time’ would be better.”* (Participant 01-001). *“…Yeah that might be another answer that, no I did not have enough time. I had to make another appointment.”* (Participant 01-005).

#### Item 17 – impact of the IV infusion on time for talking to a nurse/doctor

For item 17, six changes were suggested: lengthening the recall period (*n* = 3), eliminating the item (*n* = 1), clarifying the logic of the item question (*n* = 1), increasing the response options (*n* = 1), creating an open comment area (*n* = 1), and making an overall change to the item (*n* = 1). *“I don’t think it’s relevant, because… I don’t see where an IV infusion would impact the amount of time you have to talk to your doctor about the illness. Well, I guess ‘cause other concerns. If other concerns is related to the illness, no…. I would just change the co- question completely.”* (Participant 01-009).

#### Item 18 – treatment preference

For item 18, four changes were suggested: providing more description of the SC treatment (*n* = 5), changing the item to reflect that SC treatment is not currently available (*n* = 1), simplifying the details on the IV option (*n* = 1), and dividing IV treatment into two different options (*n* = 1). *“…the first part should be actually two...I mean, there’s a difference between getting a needle and having a port. That’s...two different things completely.”* (Participant 01-003).

#### Item 19 – recommending the IV infusion

Regarding item 19, three changes were suggested: making the item more specific (*n* = 1), adding another response option (*n* = 1), and making the item more nuanced (*n* = 1). *“That’s a difficult question. ...I put probably not ‘cause I think I was thinking about the whole...the place that I go to get it and the people that do it and ‘would I recommend them’...I’m not sure about how that’s asked or what they’re getting at... Well, the question…needs work.”* (Participant 01-005).

While suggested changes were made for all 19 items, changes to the measure were not recommended, largely because most were suggested by only *n* = 1, but also for the reasons detailed in Supplementary Table 4 (Fig. 4).

### Gaps and participant suggestions for change for TASQ-IV measure overall

When asked their opinion of the measure as a whole, six participants suggested nine types of changes to TASQ-IV, some general and some item-specific: making the items past tense (*n* = 2), providing an open-ended response option for item 2 (*n* = 1), further explaining the purpose of the questionnaire (*n* = 1), removing items 15 and 17 (both *n* = 1), lengthening the recall period (*n* = 1) (Supplementary Table 5).

When participants discussed gaps in the content of the TASQ-IV content, they suggested that questions should be added regarding “brain fog” (*n* = 1) and side effects (*n* = 1), and that questions should be inclusive to those with IV ports (*n* = 1; Supplementary Table 5).

After consideration by the authors, such changes to the measure were not recommended because each suggestion was made by only a few participants (generally *n* = 1, at most *n* = 2), and deemed not relevant to clinical trial completion.

### Participant opinions and suggestions for the TASQ-SC instructions

All 10 participants demonstrated that they understood the TASQ-SC instructions. Of the eight participants asked about the relevance of the TASQ-SC instructions, seven indicated they were relevant.

Nine participants were asked to describe their suggested changes to the TASQ-SC instructions. Five suggestions were made, although as they were each recommended by one participant only they were not implemented. The suggestions were: adding a phrase to the instructions to prevent someone from completing the TASQ-SC if they haven’t had SC treatment (*n* = 1), lengthening the recall period (*n* = 1), changing the tense of the instructions to match the questions (*n* = 1), reiterating the recall period (*n* = 1), and shortening the instructions to the last sentence only (*n* = 1; Supplementary Table 6).

### Overview of key findings

Nearly all participants demonstrated that they understood the TASQ-IV/TASQ-SC instructions and most indicated that the TASQ-IV items were clearly understandable, relevant and that the response options were suitable.

Most participants stated that a longer or more general period for recalling information might be suitable for many of the items. However, considering that the TASQ-IV is intended for use in clinical trials where only the most recent treatment will be considered, changing the recall period was deemed unnecessary.

Item 17 (impact of IV infusion on the amount of time to talk to nurse and/or doctor) was consistently reported to be of low relevance. It is possible that medical professionals in the clinic may have limited experience of PNH, which may have contributed to how participants answered this question. However, because clinical trial staff are likely to have more knowledge of PNH, limited understanding of the disease is unlikely to be an issue in clinical trial settings. Medical professionals in the clinic may also have less time to talk at length with the participants compared with clinical trial staff. IV infusion may also not affect the time available to talk to a nurse or doctor because adequate time is available for this in clinic, this being the reason for the considered low relevance of the item.

Only minor suggested changes to individual items were made by the participants. Each change was generally suggested by only one participant and considered a consequence of the one-off completion of the measure in an interview setting and was not considered to be of concern in a clinical trial. In this setting, the increased knowledge of PNH of the clinical trial staff and the opportunity for participants to be trained in the measures would ameliorate these issues. Consequently, no changes to the TASQ-IV/TASQ-SC are recommended for their use in a clinical trial.

It should also be noted that while this study demonstrates that TASQ-IV/TASQ-SC are suitable for use in a clinical trial setting, they may not necessarily always be administered in this setting, and so further research is warranted. In this respect, the participants’ suggestions in this study may provide ways in which the measure could be modified for use beyond clinical trial settings.

### Limitations

The study population consists of English-speaking white/Caucasian patients of which a greater proportion were educated to a more advanced level compared with the wider USA population; although cross-cultural and educational differences in content validity are not expected, the variety of suggestions made may reflect the efforts of the participants to engage with the research and not overall problems with the measure. PNH is a rare disease, for this reason the study sample was small; further research in a larger cross-cultural population might be beneficial. Furthermore, the interview guide was shortened so that interviews could be completed in the allotted time; this reduced the comprehensiveness of the data gathered, and saturation analysis of the data collected in the section on introduction to disease history was not possible. Finally, content validation and psychometric properties of RASQ were assessed among patients with non-Hodgkin lymphoma (NHL). While the use of rituximab for the treatment of NHL is time restricted (approximately 2 years for maintenance dosing), eculizumab is a lifelong treatment for patients with PNH [17, 19].

## Conclusions

The majority of participants indicated that they understood the TASQ-IV/TASQ-SC instructions and that the TASQ-IV items were clearly understood, relevant and the response options were suitable.

Many minor suggestions for change were made by participants to individual items of the TASQ-IV, however each suggestion was generally made by 1 or 2 participants, and was generally related to a one-off completion of the measure in an interview setting, or would not be a concern in a clinical trial setting. While all participant suggestions for changes were given serious consideration, the authors agreed that these would not be beneficial for administration of the TASQ-IV (or TASQ-SC) within a clinical trial setting.

The TASQ-IV/TASQ-SC measures are therefore considered suitable for use in a clinical trial in the PNH population.

## Supplementary Information

**Additional file 1 : Supplementary Material.** Treatment Administration Satisfaction Questionnaire – Intravenous (TASQ-IV). Treatment Administration Satisfaction Questionnaire – Subcutaneous (TASQ-SC). **Supplementary Table 1** Participant eligibility criteria. **Supplementary Table 2** Participant opinions about the TASQ-IV instructions. **Supplementary Table 3** Participant-suggested changes to TASQ-IV instructions. **Supplementary Table 4** Participant-suggested changes to TASQ-IV items. **Supplementary Table 5** Gaps and participant suggestions for change for TASQ-IV measure overall. **Supplementary Table 6** Participant-suggested changes to TASQ-SC instructions.

## Data Availability

All data generated or analyzed during this study are included in this published article and its supplementary information files.

## Abbreviations

AA-MDS: Aplastic Anemia and MDS International Foundation
COS: Clinical Outcomes Solutions
IQR: Interquartile range
IV: Intravenous
NHL: Non-Hodgkin lymphoma
PNH: Paroxysmal nocturnal hemoglobinuria
RASQ: Rituximab Administration Satisfaction Questionnaire
SC: Subcutaneous
SD: Standard deviation
TASQ: Treatment Administration Satisfaction Questionnaire
