# Supplementary material for: Concept confirmation of the Treatment Administration Satisfaction Questionnaire (TASQ) in rare paroxysmal nocturnal hemoglobinuria
Source: J Patient Rep Outcomes. 2021 Jun 21;5:45. doi: 10.1186/s41687-021-00319-9 (PMC8217362; doi:10.1186/s41687-021-00319-9)
Supplement: Supplementary file 1 — Additional file 1 : Supplementary Material. Treatment Administration Satisfaction Questionnaire – Intravenous (TASQ-IV). Treatment Administration Satisfaction Questionnaire – Subcutaneous (TASQ-SC). Supplementary Table 1 Participant eligibility criteria. Supplementary Table 2 Participant opinions about the TASQ-IV instructions. Supplementary Table 3 Participant-suggested changes to TASQ-IV instructions. Supplementary Table 4 Participant-suggested changes to TASQ-IV items. Supplementary Table 5 Gaps and participant suggestions for change for TASQ-IV measure overall. Supplementary Table 6 Participant-suggested changes to TASQ-SC instructions. [file 41687_2021_319_MOESM1_ESM.docx]

# Supplementary Material

**Treatment Administration Satisfaction Questionnaire – Intravenous (TASQ-IV)**

**Instructions**: Please complete the following questions based on your eculizumab treatment. Your eculizumab was given through a thin plastic tube and a needle that was put directly into a vein in your arm, called an intravenous or IV infusion. Please answer the questions **based on your most recent eculizumab IV infusion**.

1. Thinking about the IV infusion, how satisfied or dissatisfied are you with the IV infusion?

| Very satisfied | Satisfied | Neither satisfied nor dissatisfied | Dissatisfied | Very dissatisfied |
| --- | --- | --- | --- | --- |

2. Thinking about the IV infusion, how do you rate the pain, swelling, or redness you experienced at the site of the drug injection?

| None | Mild | Moderate | Severe | Very severe |
| --- | --- | --- | --- | --- |

3. Thinking about the IV infusion, how do you rate the pain you experience with the IV infusion process?

| None | Mild | Moderate | Severe | Very severe |
| --- | --- | --- | --- | --- |

4. Thinking about the IV infusion, are the side effects of the IV infusion as you expected?

| Much better than expected | Somewhat better than expected | Met my expectations | Somewhat worse than my expectations | Much worse than my expectations |
| --- | --- | --- | --- | --- |

5. Before you receive the IV infusion, do you feel anxious about having the infusion?

| Not at all | A little bit | Somewhat | Quite a bit | Very much |
| --- | --- | --- | --- | --- |

6. When you receive the IV infusion, do you worry that your condition would get worse?

| Not at all | A little bit | Somewhat | Quite a bit | Very much |
| --- | --- | --- | --- | --- |

7. When you receive the IV infusion, do you feel anxious thinking about your disease?

| Not at all | A little bit | Somewhat | Quite a bit | Very much |
| --- | --- | --- | --- | --- |

8. Thinking about IV infusion, how confident are you that the IV infusion is treating your disease?

| Not at all | A little bit | Somewhat | Quite a bit | Very much |
| --- | --- | --- | --- | --- |

9. When you receive the IV treatment, do you feel restricted by the IV infusion?

| Not at all | A little bit | Somewhat | Quite a bit | Very much |
| --- | --- | --- | --- | --- |

10. Thinking about the IV infusion, how convenient is it for you to get your IV infusion?

| Very convenient | Convenient | Neither convenient nor inconvenient | Inconvenient | Very inconvenient |
| --- | --- | --- | --- | --- |

11. Thinking about the IV infusion, how do you feel about the amount of time it takes to get your IV infusion?

| Too short | Just right | Too long |  |  |
| --- | --- | --- | --- | --- |

12. Thinking about the IV infusion, do you feel that the length of time to get your IV infusion was as you expected?

| Much shorter than expected | Somewhat shorter than expected | As expected | Somewhat longer than expected | Much longer than expected |
| --- | --- | --- | --- | --- |

13. Thinking about the IV infusion, how bothered are you by the amount of time it takes to get the infusion?

| Not at all bothered | A little bothered | Moderately bothered | Quite bothered | Very bothered |
| --- | --- | --- | --- | --- |

14. How much does the IV infusion:

a) Interfere with your usual or daily activities?

| Not at all | A little bit | Somewhat | Quite a bit | Very much |
| --- | --- | --- | --- | --- |

b) Limit your daily activities?

| Never | Rarely | Sometimes | Most of the time | Always |
| --- | --- | --- | --- | --- |

15. Because of the length of time to apply the IV infusion, do you feel that you have lost or gained time for other things?

| Lost a lot of time | Lost some time | Neither lost nor gained time | Gained some time | Gained a lot of time |
| --- | --- | --- | --- | --- |

16. When you receive the IV infusion treatment, are you able to talk to your nurse and/or doctor as much as you would like about your illness? (please only check ONE answer)

| ⁮ | Yes, I had more than enough time to talk to my nurse and/or doctor. |
| --- | --- |
| ⁮ | Yes, but I would have liked more time to talk to my nurse and/or doctor. |
| ⁮ | It does not matter to me if I have time to talk to my nurse and/or doctor during my treatment. |
| ⁮ | No, I did not have enough time to talk to my nurse and/or doctor. |
| ⁮ | No, I did not talk to my nurse and/or doctor at all. |

17. Does the IV infusion impact the amount of time you have to talk to your nurse and/or doctor about your illness and other concerns?

| Yes | No |  |  |  |
| --- | --- | --- | --- | --- |

18. Thinking about the IV infusion, if given the option, which would you prefer (both options treat your disease in the same way)? Please check one.

| - Prefer intravenous (IV) injection given through a port or a thin plastic tube and a needle into your vein (IV drip). This treatment option usually takes 0.5 to 2 hours. |
| --- |
| - Prefer subcutaneous (SC) injection, applied with a device on the thigh or abdomen (or belly).  This treatment option usually takes 10 to 30 minutes. |
| - No preference for treatment option. |

19. Thinking about the IV infusion, would you recommend the way you received the treatment (IV infusion) to another patient?

| Definitely yes | Probably yes | I don’t know | Probably not | Definitely not |
| --- | --- | --- | --- | --- |

**Treatment Administration Satisfaction Questionnaire – Subcutaneous (TASQ-SC)**

**Instructions**: Please complete the following questions based on your ravulizumab* treatment. Your ravulizumab* was given through a device placed onto your, thigh, or abdomen (or belly) area, called a subcutaneous or SC infusion. Please answer the questions **based on your most recent ravulizumab* SC infusion**.

1. Thinking about the SC infusion, how satisfied or dissatisfied are you with the SC infusion?

| Very satisfied | Satisfied | Neither satisfied nor dissatisfied | Dissatisfied | Very dissatisfied |
| --- | --- | --- | --- | --- |

2. Thinking about the SC infusion, how do you rate the pain, swelling, or redness you experienced at the site of the drug infusion?

| None | Mild | Moderate | Severe | Very severe |
| --- | --- | --- | --- | --- |

3. Thinking about the SC infusion, how do you rate the pain you experience with the SC infusion process?

| None | Mild | Moderate | Severe | Very severe |
| --- | --- | --- | --- | --- |

4. Thinking about the SC infusion, are the side effects of the SC infusion as you expected?

| Much better than expected | Somewhat better than expected | Met my expectations | Somewhat worse than my expectations | Much worse than my expectations |
| --- | --- | --- | --- | --- |

5. Before you receive the SC infusion, do you feel anxious about having the infusion?

| Not at all | A little bit | Somewhat | Quite a bit | Very much |
| --- | --- | --- | --- | --- |

6. When you receive the SC infusion, do you worry that your condition would get worse?

| Not at all | A little bit | Somewhat | Quite a bit | Very much |
| --- | --- | --- | --- | --- |

7. When you receive the SC infusion, do you feel anxious thinking about your disease?

| Not at all | A little bit | Somewhat | Quite a bit | Very much |
| --- | --- | --- | --- | --- |

8. Thinking about SC infusion, how confident are you that the SC infusion is treating your disease?

| Not at all | A little bit | Somewhat | Quite a bit | Very much |
| --- | --- | --- | --- | --- |

9. When you receive the SC treatment, do you feel restricted by the SC infusion?

| Not at all | A little bit | Somewhat | Quite a bit | Very much |
| --- | --- | --- | --- | --- |

10. Thinking about the SC infusion, how convenient is it for you to get your SC infusion?

| Very convenient | Convenient | Neither convenient nor inconvenient | Inconvenient | Very inconvenient |
| --- | --- | --- | --- | --- |

11. Thinking about the SC infusion, how do you feel about the amount of time it takes to get your SC infusion?

| Too short | Just right | Too long |  |  |
| --- | --- | --- | --- | --- |

12. Thinking about the SC infusion, do you feel that the length of time to get your SC infusion was as you expected?

| Much shorter than expected | Somewhat shorter than expected | As expected | Somewhat longer than expected | Much longer than expected |
| --- | --- | --- | --- | --- |

13. Thinking about the SC infusion, how bothered are you by the amount of time it takes to get the infusion?

| Not at all bothered | A little bothered | Moderately bothered | Quite bothered | Very bothered |
| --- | --- | --- | --- | --- |

14. How much does the SC infusion:

a) Interfere with your usual or daily activities?

| Not at all | A little bit | Somewhat | Quite a bit | Very much |
| --- | --- | --- | --- | --- |

b) Limit your daily activities?

| Never | Rarely | Sometimes | Most of the time | Always |
| --- | --- | --- | --- | --- |

15. Because of the length of time to apply the SC infusion, do you feel that you have lost or gained time for other things?

| Lost a lot of time | Lost some time | Neither lost nor gained time | Gained some time | Gained a lot of time |
| --- | --- | --- | --- | --- |

16. When you receive the SC infusion treatment, are you able to talk to your nurse and/or doctor as much as you would like about your illness? (please only check ONE answer)

| ⁮ | Yes, I had more than enough time to talk to my nurse and/or doctor. |
| --- | --- |
| ⁮ | Yes, but I would have liked more time to talk to my nurse and/or doctor. |
| ⁮ | It does not matter to me if I have time to talk to my nurse and/or doctor during my treatment. |
| ⁮ | No, I did not have enough time to talk to my nurse and/or doctor. |
| ⁮ | No, I did not talk to my nurse and/or doctor at all. |

17. Does the SC infusion impact the amount of time you have to talk to your nurse and/or doctor about your illness and other concerns?

| Yes | No |  |  |  |
| --- | --- | --- | --- | --- |

18. Thinking about the SC treatment, if given the option, which would you prefer (both options treat your disease in the same way)? Please check one.

| - Prefer intravenous (IV) injection given through a port or a thin plastic tube and a needle into your vein (IV drip). This treatment option usually takes 0.5 to 2 hours. |
| --- |
| - Prefer subcutaneous (SC) injection, applied with a device on the thigh or abdomen (or belly).  This treatment option usually takes 10 to 30 minutes. |
| - No preference for treatment option. |

19. Thinking about the SC treatment, would you recommend the way you received the treatment (SC infusion) to another patient?

| Definitely yes | Probably yes | I don’t know | Probably not | Definitely not |
| --- | --- | --- | --- | --- |

*The version of the TASQ-SC that the participants reviewed referred to eculizumab (Soliris) rather than ravulizumab (Ultomiris). This was because at the time of interview ravulizumab was not approved by the FDA. This does not influence the findings as it is not relevant to the evaluation of the questionnaire content.

**Supplementary Table 1** Participant eligibility criteria

| **Inclusion criteria** | **Exclusion criteria** |
| --- | --- |
| - Male or female aged ≥ 18 years at the time of providing informed consent - Body weight from 40 kg to 100 kg - PNH diagnosis confirmed by qualified healthcare professional - Current treatment of PNH with Soliris - Willing and able to give written informed consent and comply with study interview procedures | - History of bone marrow transplantation or *Neisseria meningitis* infection - History of unexplained infections - HIV - History of malignancy within 5 years of screening, except for nonmelanoma skin cancer or carcinoma in situ of the cervix that has been treated with no evidence of recurrence - Females who are currently pregnant or breastfeeding - Known medical or psychological condition(s) or risk factor that, in the opinion of the Investigator, might interfere with the patient’s full participation in the study, pose an additional risk for the patient, or confound the outcome of the study - Unstable medical conditions that would make them unlikely to tolerate the requirements of the protocol |

*HIV* human immunodeficiency virus, *PNH*, paroxysmal nocturnal hemoglobinuria

**Supplementary Table 2** Participant opinions about the TASQ-IV instructions

| **TASQ-IV Debrief** | **Number of participants (*N* = 10)** | | |
| --- | --- | --- | --- |
|  | **Understanding (*n* = 10)** | **Relevance (*n* = 10)** | **Suggestions for change (*n* = 10)** |
| Yes | 9 | 9 | 2 |
| Maybe | 1 | 1 | 1 |
| No | 0 | 0 | 7 |

*TASQ-IV* Treatment Administration Satisfaction Questionnaire – Intravenous

**Supplementary Table 3** Participant-suggested changes to TASQ-IV instructions

| **Suggested change** | **No. of participants** | **Decision** |
| --- | --- | --- |
| Adding a port option to the instructions | 1 | No change to the questionnaire is required because this was only suggested by one participant and is not critical to the clinical trial. |
| Omitting detailed description of the IV treatment* | 1 | No change to the questionnaire is required because this was only suggested by one participant and keeping the details ensures understanding for those that need it. |
| Mentioning subcutaneous treatment as an option | 1 | No change to the questionnaire is required because this was only suggested by one participant. |

*IV* intravenous; *TASQ* Treatment Administration Satisfaction Questionnaire

*The participant’s response regarding their suggestion for change was ‘maybe’.

**Supplementary Table 4** Participant-suggested changes to TASQ-IV items

| **TASQ-IV item number** | **Suggested change** | **Number of participants** | **Decision** |
| --- | --- | --- | --- |
| 1 | Lengthening the recall period | 1 | No change to the questionnaire item is required.  In a clinical setting, patients would be filling this form regularly to detect a change from treatment to treatment. |
| 1 | Teasing out what satisfaction and dissatisfaction entails | 1 | No change to the questionnaire item is required.  The item asks for a general impression. The TASQ-IV already has specific questions about different aspects of the treatment method. |
| 2 | Expanding the body area from injection site to whole arm | 1 | No change to the questionnaire item is required.  This was only suggested by one participant. Also, the focus of the question is the injection site. |
| 2 | Measuring redness, pain and swelling separately | 1 | No change to the questionnaire item is required.  This was only suggested by one participant. |
| 3 | Changing the item into past tense | 1 | No change to the questionnaire item is required.  This was only suggested by one participant. While this suggestion is reasonable, it is not fundamental to the clinical trial. |
| 3 | Differentiating between the IV infusion process from experience afterward | 1 | No change to the questionnaire item is required.  The item already states “the IV infusion process.” |
| 3 | Lengthening the recall period | 1 | No change to the questionnaire item is required.  If used in a population, not in a clinical trial, then the time period could be extended. However, in a clinical trial setting, the time frame is suitable. |
| 3 | Mentioning recall period in the item | 1 | No change to the questionnaire item is required. When used in a clinical trial setting where this is administered after a treatment then the time frame is suitable. |
| 4 | Changing the item into past tense | 1 | No change to the questionnaire item is required.  This was only suggested by one participant. While this suggestion is reasonable, it is not fundamental to the clinical trial. |
| 4 | Adding the word “process” after the IV infusion | 1 | No change to the questionnaire item is required.  This was only suggested by one participant. |
| 4 | Differentiating the first IV experience from the last IV experience* | 1 | No change to the questionnaire item is required.  This was only suggested by one participant. |
| 4 | Comparing severity of side effects to any physician described expectations | 1 | No change to the questionnaire item is required.  This was only suggested by one participant. |
| 4 | Lengthening the recall period | 1 | No change to the questionnaire item is required.  If used in a population not in a clinical trial, then the time period could be extended. However, in a clinical trial setting where meaningful change is measured, the time frame is suitable. |
| 4 | Adding possible side effects | 1 | No change to the questionnaire item is required.  This was only suggested by one participant. |
| 5 | Changing the item into past tense | 1 | No change to the questionnaire item is required.  This was only suggested by one participant. While this suggestion is reasonable, it is not fundamental to the clinical trial. |
| 5 | Lengthening the recall period* | 1 | No change to the questionnaire item is required.  If used in a population, not in a clinical trial, then the time period could be extended. However, in a clinical trial setting where meaningful change is measured, the time frame is suitable. |
| 6 | Changing the item to reflect that the drug itself is not making the condition worse | 2 | No change to the questionnaire item is required. The item is not making a connection between condition getting worse and Soliris. |
| 6 | Differentiating the IV process from the drug itself | 1 | No change to the questionnaire item is required.  The item is asking general sense of worry about deterioration of health, thus there is no need to differentiate between the IV process and the drug itself. |
| 6 | Lengthening the recall period | 1 | No change to the questionnaire item is required. If used in a population, not in a clinical trial, then the time period could be extended. However, in a clinical trial setting where meaningful change is measured, the time frame is suitable. |
| 6 | Broadening the item question to ask about overall health instead of just PNH (“condition”) | 1 | No change to the questionnaire item required.  Suggested by only one participant. |
| 7 | Differentiating the IV process from the drug itself | 1 | No change to the questionnaire item is required.  The item is asking a general sense of anxiousness about the disease, thus there is no need to differentiate between the IV process and the drug itself. |
| 7 | Changing the question into past tense | 1 | No change to the questionnaire item is required.  This was only suggested by one participant. While this suggestion is reasonable, it is not fundamental to the clinical trial. |
| 8 | Changing the question into past tense | 1 | No change to the questionnaire item is required.  This was only suggested by one participant. While this suggestion is reasonable, it is not fundamental to the clinical trial. |
| 8 | Lengthening the recall period | 1 | No change to the questionnaire item is required.  If used in a population, not in a clinical trial, then the time period could be extended. However, in a clinical trial setting the time frame is suitable. |
| 8 | Clarifying whether the item is about the treatment or the drug | 1 | No change to the questionnaire item is required.  The suggestion is not clear but inferred. |
| 8 | Stating the name of the drug in the item question | 1 | No change to the questionnaire item is required.  This was only suggested by one participant and the drug in the context is obvious to participants. |
| 8 | Mentioning “confident” in each response option | 1 | No change to the questionnaire item is required.  This was only suggested by one participant. |
| 8 | Ask recall “before treatment” | 1 | No change to the questionnaire item is required.  Idiosyncratic. Change will be measured via multiple assessments *after* treatment. |
| 9 | Changing the item into past tense | 1 | No change to the questionnaire item is required.  This was only suggested by one participant. While this suggestion is reasonable, it is not fundamental to the clinical trial. |
| 9 | Making the item more specific | 1 | No change to the questionnaire item is required.  This was only suggested by one participant. |
| 9 | Clarifying the exact time frame of restriction (during the infusion vs. day of infusion)* | 1 | No change to the questionnaire item is required.  The item already specifies the time frame by stating “When you receive the IV treatment.” |
| 9 | Specifying the type of restriction* | 1 | No change to the questionnaire item is required.  This was only suggested by one participant. |
| 9 | Having response options refer to the item | 1 | No change to the questionnaire item is required.  This was only suggested by one participant. |
| 10 | Separating home infusions from the clinic ones | 2 | No change to the questionnaire item is required. The sponsor is already taking account of participants who are getting treatment at home and/or clinic and the trial participants will complete the questionnaire with reference to where the infusion was made. |
| 10 | Taking out one of the response options (i.e. inconvenient) | 1 | No change to the questionnaire item is required. Suggested by only one participant. |
| 10 | Clarifying the time frame (i.e. drip time vs. total visit time) | 1 | No change to the questionnaire item is required.  This concern is likely to reflect the one-off completion of the measure in an interview setting. If given regularly during a clinical trial, the meaning would be clear, and this would not be an issue. |
| 10 | Combine items 9 (*restriction by IV infusion*) and 10 (*convenience of IV infusion*) | 1 | No change to the questionnaire item is required.  Suggested only by one participant; the items are intended to ask about these concepts separately. |
| 11 | Clarifying the time period regarding the IV infusion (i.e. during infusion vs. whole time between infusions) | 4 | No change to the questionnaire item is required.  This concern is likely to reflect the one-off completion of the measure in an interview setting. If given regularly during a clinical trial, the meaning would be clear, and this would not be an issue. |
| 11 | Eliminating one of the response options (i.e. too short) | 3 | No change to the questionnaire item is required.  The response option “Too short’ may not apply to some participants but it may apply to other participants (e.g. retired people). |
| 11 | Changing the item question into past tense | 1 | No change to the questionnaire item is required.  This was only suggested by one participant. While this suggestion is reasonable, it is not fundamental to the clinical trial. |
| 11 | Eliminating the item | 1 | No change to the questionnaire item is required.  This was only suggested by one participant. |
| 12 | Clarifying the time period regarding the IV infusion (i.e. during infusion vs. entire visit/travel time) | 2 | No change to the questionnaire item is required.  This concern is likely to reflect the one-off completion of the measure in an interview setting. If given regularly during a clinical trial, the meaning would be clear, and this would not be an issue. |
| 13 | Clarifying the time period regarding the IV infusion (i.e. during infusion vs. whole time between infusions) | 3 | No change to the questionnaire item is required.  This concern is likely to reflect the one-off completion of the measure in an interview setting. If given regularly during a clinical trial, the meaning would be clear, and this would not be an issue. |
| 13 | Lengthening the recall period | 1 | No change to the questionnaire item is required.  If used in a population, not in a clinical trial, then the time period could be extended. However, in a clinical trial setting where meaningful change is measured, the time frame is suitable. |
| 14A | Clarifying the time period regarding the IV infusion (i.e. during infusion vs. whole time between infusions) | 3 | No change to the questionnaire item is required. This concern is likely to reflect the one-off completion of the measure in an interview setting. If given regularly during a clinical trial, the meaning would be clear, and this would not be an issue. |
| 14A | Changing the question into past tense | 1 | No change to the questionnaire item is required. This was only suggested by one participant. While this suggestion is reasonable, it is not fundamental to the clinical trial. |
| 14A | Differentiating Item 14A from 14B more clearly | 1 | No change to the questionnaire item is required. This was only suggested by one participant. |
| 14A | Lengthening the recall period | 1 | No change to the questionnaire item is required. If used in a population, not in a clinical trial, then the time period could be extended. However, in a clinical trial setting where meaningful change is measured, the time frame is suitable. |
| 14A | Clarifying the item question | 1 | No change to the questionnaire item is required. The suggestion is not clear. |
| 14B | Clarifying the time period regarding the IV infusion (i.e. during infusion vs. whole time between infusions) | 2 | No change to the questionnaire item is required.  This concern is likely to reflect the one-off completion of the measure in an interview setting. If given regularly during a clinical trial, the meaning would be clear, and this would not be an issue. |
| 14B | Eliminating the item | 2 | No change to the questionnaire item is required.  This was only suggested by two participants. |
| 14B | Lengthening the recall period | 2 | No change to the questionnaire item is required.  If used in a population, not in a clinical trial, then the time period could be extended. However, in a clinical trial setting where meaningful change is measured, the time frame is suitable. |
| 14B | Clarify the response option “Always” | 1 | No change to the questionnaire item is required.  This was only suggested by one participant. |
| 14B | Adding recall period to the item | 1 | No change to the questionnaire item is required.  This was only suggested by one participant. |
| 15 | Clarifying the time period regarding the IV infusion (i.e. during infusion vs. whole time between infusions) | 2 | No change to the questionnaire item is required.  This concern is likely to reflect the one-off completion of the measure in an interview setting. If given regularly during a clinical trial, the meaning would be clear, and this would not be an issue. |
| 15 | Changing a word to make it easier to read | 1 | No change to the questionnaire item is required.  This was only suggested by one participant. |
| 15 | Asking for longer recall in addition to the last treatment | 1 | No change to the questionnaire item is required.  This was only suggested by one participant. |
| 15 | Changing a phrase to make it easier to read* | 1 | No change to the questionnaire item is required.  This was only suggested by one participant. |
| 15 | Getting rid of one of the options | 1 | No change to the questionnaire item is required.  This was only suggested by one participant. |
| 15 | Making the item more nuanced | 1 | No change to the questionnaire item is required.  This was only suggested by one participant. |
| 16 | Adding new response options | 2 | No change to the questionnaire item is required.  Adding new response options were discussed by two participants, but they were two different suggestions. Therefore, it is likely to be idiosyncratic and should not be considered in the measure. |
| 16 | Lengthening the recall period | 1 | No change to the questionnaire item is required. If used in a population, not in a clinical trial, then the time period could be extended. However, in a clinical trial setting where meaningful change is measured, the time frame is suitable. |
| 16 | Shortening response option statements | 1 | No change to the questionnaire item is required.  This was only suggested by one participant. |
| 16 | Taking the word “nurse” out of the item question and response options | 1 | No change to the questionnaire item is required.  This was only suggested by one participant. |
| 16 | Separating the item question for each nurse and doctor | 1 | No change to the questionnaire item is required.  This was only suggested by one participant. |
| 16 | Change the item question to ask about the IV process and not illness | 1 | No change to the questionnaire item is required.  This was only suggested by one participant. |
| 16 | Clarify the item* | 1 | No change to the questionnaire item is required.  This was only suggested by one participant. |
| 17 | Lengthening the recall period | 3 | No change to the questionnaire item is required.  If used in a population, not in a clinical trial, then the time period could be extended. However, in a clinical trial setting where meaningful change is measured, the time frame is suitable. |
| 17 | Eliminating the item | 1 | No change to the questionnaire item is required.  This was only suggested by one participant. |
| 17 | The item needs overall change | 1 | No change to the questionnaire item is required.  This was only suggested by one participant. |
| 17 | Clarifying the logic of the item question | 1 | No change to the questionnaire item is required.  This was only suggested by one participant. |
| 17 | Increase the response options | 1 | No change to the questionnaire item is required.  This was only suggested by one participant. |
| 17 | Creating an open comment area | 1 | No change to the questionnaire item is required.  This was only suggested by one participant. |
| 18 | Providing more description of the SC treatment | 5 | No change to the questionnaire item is required.  In a clinical setting, participants will know the context in which the SC treatment will be applied. |
| 18 | Changing item to reflect that the SC treatment is not available at the moment | 1 | No change to the questionnaire item is required.  In a clinical trial setting, the SC treatment would be available, and participants would know more about it. |
| 18 | Dividing the IV treatment to two different options | 1 | No change to the questionnaire item is required.  This was only suggested by one participant. |
| 18 | Simplifying the details on the IV option | 1 | No change to the questionnaire item is required. This was only suggested by one participant. |
| 19 | The item could be more specific* | 1 | No change to the questionnaire item is required.  This was only suggested by one participant. |
| 19 | Making the item more nuanced* | 1 | No change to the questionnaire item is required.  This was only suggested by one participant. |
| 19 | Adding another response option | 1 | No change to the questionnaire item is required.  This was only suggested by one participant. |

*IV* intravenous, *PNH* paroxysmal nocturnal hemoglobinuria, *SC* subcutaneous, *TASQ* Treatment Administration Satisfaction Questionnaire

*The participant’s response regarding their suggestion for change was ‘maybe’.

**Supplementary Table 5** Participant-suggested changes to TASQ-IV overall measure

| **Suggested change** | **No. of participants** | **Decision** |
| --- | --- | --- |
| Making the items past tense | 2 | No change to the questionnaire is required.  While this suggestion is reasonable, it is not fundamental to the clinical trial. |
| Give an open-ended response option for Item 2 | 1 | No change to the questionnaire is required.  This change was suggested by only one participant. |
| The purpose of the questionnaire needs to be better explained | 1 | No change to the questionnaire is required.  This is due to the one-off completion of the measure in an interview setting. If given regularly during a clinical trial, this would be clear. |
| Removing Item 17 | 1 | No change to the questionnaire is required.  This change was suggested by only one participant. |
| Removing Item 15 | 1 | No change to the questionnaire is required.  This change was suggested by only one participant. |
| Lengthening the recall period | 1 | No change to the questionnaire is required.  This is due to the one-off completion of the measure in an interview setting. |
| Asking about different symptoms of the disease | 1 | No change to the questionnaire is required.  This change was suggested by only one participant. |
| Asking about side effects | 1 | No change to the questionnaire is required.  This change was suggested by only one participant. |
| Keep people with ports into consideration | 1 | No change to the questionnaire is required.  This change was suggested by only one participant. |

*IV* intravenous, *TASQ* Treatment Administration Satisfaction Questionnaire

**Supplementary Table 6** Participant-suggested changes to TASQ-SC instructions

| **Suggested change*** | **No. of participants (*N* = 4)** | **Decision** |
| --- | --- | --- |
| Adding a phrase to instructions to prevent someone from taking TASQ-SC if they have not had subcutaneous treatment | 1 | No change to the questionnaire is required.  This should not be an issue in a clinical setting where the SC form of treatment is administered. |
| Lengthening the recall period | 1 | No change to the questionnaire is required.  Considering the context of use for the TASQ instruments, the recall is appropriate. |
| Change tense of the instructions to match the questions | 1 | No change to the questionnaire is required.  While this suggestion is reasonable, it is not fundamental to the clinical trial. The change is optional. |
| Reiterate recall period | 1 | No change to the questionnaire is required.  The instructions already state the recall period. |
| Shorten instructions to last sentence only (i.e. “*Please answer the questions based on your most recent Ultomiris** SC infusion”*) | 1 | No change to the questionnaire is required.  This is suggested by one participant only and did not prevent understanding of the instructions. |

*SC* subcutaneous, *TASQ* Treatment Administration Satisfaction Questionnaire

*Question of suggested changes was not explicitly asked to one participant.

**The version of the TASQ-SC that the participants reviewed referred to eculizumab (Soliris) rather than ravulizumab (Ultomiris). This was because at the time of interview ravulizumab was not approved by the FDA. This does not influence the findings as it is not relevant to the evaluation of the questionnaire content.
